# Supplementary material for: PyHIST: A Histological Image Segmentation Tool
Source: PLoS Comput Biol. 2020 Oct 19;16(10):e1008349. doi: 10.1371/journal.pcbi.1008349 (PMC7647117; doi:10.1371/journal.pcbi.1008349)
Supplement: S1 Text — General description of the pipeline: supported file formats, tile generation methods, and execution times. (PDF) [file pcbi.1008349.s001.pdf]

Supplementary Text

S1: PyHIST overview

S1.1 Overview of PyHIST

PyHIST is a high-resolution histological image segmentation pipeline. It takes as input a Whole Slide Image (WSI) encoded in Aperio SVS/TIFF format (Fig 1a) and produces a series of image tiles (Fig 1e), with dimensions specified by the user. Experimental support is provided for the following formats: Trestle (.tif), Hamamatsu (.ndmi, .vms). Square image tiles are extracted from the regions of the WSI that have tissue content (foreground) above a given threshold with respect to the total area of the tile, although it is also possible to extract all the tiles from the WSI, including those that are composed of background. Tile size is set through the *--patch-size* flag. Tile extraction is performed as if overlaying a grid with tiles of a fixed size over the original image (Fig 1d), and then keeping the relevant tiles. Tiles can be saved in JPG or PNG formats. Either the original resolution of the image or a downsampled version can be used to generate the tiles. When the process is finished, a tab-delimited text file will also be produced indicating zero-based tile coordinates in the grid and the size of each tile in pixels, as well as an indicator column stating if the tile passed the content threshold to be considered foreground.

S1.2 Tile generation methods

Although it is possible to perform the segmentation at the highest resolution encoded in the WSI, by default PyHIST will output tiles from a downsampled version (i.e. a lower resolution version of the image, scaled by a factor). The user can specify a downsampling factor (powers of 2) to perform tile extraction at a lower resolution (see argument *--output-downsample*, S2 Text). Of note, incomplete tiles (i.e. tiles that do not meet the requested tile size) can be generated towards the right and lower borders of the image due to the selected tile size not being an exact multiple of the WSI width and/or height. In this case, the size of these tiles will be as large as possible, without exceeding the specified tile dimension. Note that by default, PyHIST will not save the tiles to disk. This is to avoid a large number of files being generated by mistake when working with WSIs. The *--save-patches* flag has to be explicitly defined when launching PyHIST in order to save the tiles.

PyHIST uses graph-based segmentation as the default method to generate the mask, since we found it to be the most robust method for different types of use cases. Three alternative methods for tile generation are also available: Otsu thresholding, adaptive thresholding and random sampling. Here, we present an overview of each method.

Graph-based segmentation

In order to identify the sections of the WSI that contain tissue, PyHIST first applies a Canny edge detector [1] to generate a version of the image that highlights edges within tissue fragments. This image is then used in combination with a graph segmentation algorithm [2] to select connected components in the image, obtaining a clean mask that corresponds to tissue segments (Fig 1c). The mask will be then divided into tiles. Each tile will be inspected to determine if it's composed by at least a certain percentage of tissue content with respect to the total area of the tile (see argument *--content-threshold*, S2 Text). If the tile is kept, then the corresponding area of the original WSI will be retrieved.

Several options are available for graph-based segmentation in order to tune PyHIST for specific use-cases. Commonly, tissues are placed towards the center of the slide during the imaging process. However, in some cases, tissue can be located in the borders of the slide, and depending on the application, the user might want to keep or remove these regions. PyHIST provides parameters to deal with these types of cases (S2 Text).

A test mode (see argument *--method "graphtestmode"*) is available for graph-based segmentation in order to inspect how a mask will look with a given combination of segmentation parameters, as well as to verify the tiling grid that will be generated at the selected tile dimensions (S2 Fig). This is to aid the user in inspecting the output before producing the individual tile files. When the test mode is invoked, no tile selection will be performed, as it only serves to assess how the tile grid will be generated.

Otsu and adaptive thresholding

Two thresholding-based methods are available in PyHIST to generate the input image mask: Otsu (S3c Fig) and adaptive thresholding (S3d Fig). The input image is first converted into grayscale, and then a Gaussian kernel is applied to remove noise. The pixels in the image should then have a roughly bimodal distribution. In the case of Otsu thresholding, a value between the two peaks in the pixel distribution is calculated as the thresholding point, while with the adaptive method, the

threshold value will be calculated within small regions of the image using a weighted sum of neighbouring pixels. We find that the graph-based tiling method (S3e Fig) has a more robust performance when dealing with complex WSIs (for example, breast and adipose tissues, where parts of the tissue could be easily mistaken as background) and is thus, the default method used by PyHIST; but thresholding-based methods have the advantage of being parameter-free in PyHIST and tend to work well in tissues with homogeneous composition.

Random sampling

With random sampling mode, the user can extract tiles from random starting positions in the WSI. Content thresholding is not performed in this mode: tiles are generated directly from the randomly sampled positions. The number of tiles to sample is controlled by the *--npatches* flag. This mode can be useful to generate random data to perform sanity checks when constructing a model, since a large number of tiles might be composed of background, depending on the composition of the WSI.

S1.3 Intermediate scaling

To speed up the segmentation process, the input image (S1a Fig) can be downscaled at different resolutions, at different steps of the pipeline, not only initially. If the mask is downscaled (S1b Fig), the edge generation and segmentation process will be performed at a lower resolution, reducing the time needed to complete the process. The output resolution used to generate the tiles (S1c Fig) is independent of all other choices: for example, a user can decide to generate the mask at 16x, but output tiles at native (1x) or any other resolution. If the mask is closer to the native resolution, a more precise segmentation/thresholding is obtained, however, for most applications, such level of detail is not necessary. The same is applicable for the segmentation overview image (S1d Fig), which can be generated at any resolution independently of the other choices. All these arguments are available in the "Downsampling" section (S2 Text).

S1.4 Execution times

Random sampling

Using the WSI in S1 Fig with native dimensions 47807x38653, we performed random sampling of a varying number of tiles at different downsampling factors (S4a Fig), observing a linear trend in runtime with respect to the number of tiles. Efficiency will be determined by the resolution levels natively encoded in the WSI generation process: for this WSI, 1x, 4x and 16x are available. If the requested downsampling factors are available in the pyramidal image layers of the WSI file,

segmentation will be faster. On the other hand, if the requested downsampling factor is not encoded in the WSI (like 8x in this example), sampling will run for a longer time since resizing operations need to be performed. We also examined the runtime of performing random sampling of 1000 tiles at a fixed tile size, and at different downsampling levels (S4b Fig). As expected, runtime increases with tile size. Similarly to the previous benchmark, here we also observe the effect that the native encodings in the WSI have on the runtime speed: since 8x is not encoded in the WSI, resizing operations need to be performed, leading to longer runtimes when compared to 1x and 4x.

Graph-based segmentation

To benchmark the segmentation process, we use 50 different WSIs of stomach tissue (with different dimensions) from the Genotype-Tissue Expression (GTEx) project [3] and perform segmentation at different downsampling factors at a fixed tile size of 256x256. Runtime variability within a single downsampling factor will be determined by slide size; and segmentation runtime decreases almost linearly with respect to resolution (S4c Fig), while the runtime will increase linearly with the dimension of the slide (S4d Fig).

Otsu and adaptive thresholding

We benchmarked runtimes of Otsu and adaptive thresholding vs. graph-based segmentation for the skin WSI (GTEX-1117F-0126) shown in Fig 1a. Tile extraction was evaluated for the three different methods at four different settings of tile size: 64x64, 128x128, 256x256 and 512x512, at an output downsample of 16x and a content threshold of 0.1, explicitly saving the tiles and the tilecrossed evaluation image each time. Masks were not saved. Each method + tile size combination was repeated ten times to show runtime variability (S5 Fig). We find that Otsu and adaptive thresholding methods have smaller runtimes when compared to graph segmentation, at the expense of precision, as discussed in section S1.2.

Note: References are listed in S3 Text.
