# Supplementary material for: PyHIST: A Histological Image Segmentation Tool
Source: PLoS Comput Biol. 2020 Oct 19;16(10):e1008349. doi: 10.1371/journal.pcbi.1008349 (PMC7647117; doi:10.1371/journal.pcbi.1008349)
Supplement: S2 Text — Description of supported arguments in PyHIST. (PDF) [file pcbi.1008349.s002.pdf]

Supplementary Text

Section S2: Parameter description

S2.1 Parameters

A description for PyHIST's parameters can be obtained when launching the pipeline with the --*help* flag. The parameters in PyHIST are divided into five main functional blocks:

```
positional arguments:
  input_image           The whole slide image input file

optional arguments:
  -h, --help            show this help message and exit

Execution:
  --patch-size PATCH_SIZE
                        Integer indicating the size of the produced tiles. A
                        value of P will produce tiles of size P x P. (default:
                        512)
  --method {randomsampling,graph,graphtestmode,otsu,adaptive}
                        Method to perform the segmentation. (default: graph)
  --format {png,jpg}    Format to save the tiles. (default: png)
  --content-threshold CONTENT_THRESHOLD
                        Threshold parameter indicating the proportion of the
                        tile area that should be foreground (tissue content)
                        in order to be selected. It should range between 0 and
                        1. Not applicable for random sampling. (default: 0.5)
  --info {silent,default,verbose}
                        Show status messages at each step of the pipeline.
                        (default: default)

General output:
  --output OUTPUT       Output directory (default: output/)
  --save-patches        Save the tiles with an amount of foreground above the
                        content threshold. (default: False)
  --save-blank          If enabled, background tiles will be saved (i.e. those
                        that did not meet the content threshold.). (default:
                        False)
  --save-nonsquare      By default, only square tiles are saved, discarding
                        the regions towards the edges of the WSI that do not
                        fit a complete tile. If this flag is enabled, these
                        non-square tiles will be saved as well. (default:
```

```
                        False)
  --save-tilecrossed-image
                        Produce a thumbnail of the original image, in which
                        the selected tiles are marked with a cross. (default:
                        False)
  --save-mask           Keep the mask used to perform tile selection.
                        (default: False)

Downsampling:
  --output-downsample OUTPUT_DOWNSAMPLE
                        Downsampling factor for the output image. Must be a
                        power of 2. (default: 16)
  --mask-downsample MASK_DOWNSAMPLE
                        Downsampling factor to calculate the image mask. A
                        higher number will speed up the tiling evaluation
                        process at the expense of tile evaluation quality.
                        Must be a power of 2. (default: 16)
  --tilecross-downsample TILECROSS_DOWNSAMPLE
                        Downsampling factor to generate the tilecrossed
                        overview image. Must be a power of 2. (default: 16)
  --test-downsample TEST_DOWNSAMPLE
                        Downsampling factor to generate the test image in
                        graph test mode. Must be a power of 2. (default: 16)

Random sampling:
  --npatches NPATCHES   Number of tiles to extract in random sampling mode.
                        (default: 100)

Graph segmentation:
  --save-edges          Keep the image produced by the Canny edge detector.
                        (default: False)
  --borders
{1001,1111,0011,0101,0010,0000,0100,0110,1000,1100,1110,0001,1101,1010,1011,0111}
                        A four digit string. Each digit represents a border of
                        the image in the following order: left, bottom, right,
                        top. If the digit is 1, then the corresponding border
                        will be taken into account to define background. For
                        instance, with 1010 the algorithm will look at the
                        left and right borders of the segmented image, in a
                        window of width defined by the --percentage-bc
                        argument, and every segment identified will be set as
                        background. This argument is mutually exclusive with
                        --corners. If --borders is set to be different from
                        0000, then --corners must be 0000. (default: 1111)
```

```
  --corners
{1001,1111,0011,0101,0010,0000,0100,0110,1000,1100,1110,0001,1101,1010,1011,0111}
                        A four digit string. Each digit represents a corner of
                        the image in the following order: top left, bottom
                        left, bottom right, top right. If the digit is equal
                        to 1, then the corresponding corner will be taken into
                        account to define background. For instance, with 0101,
                        the bottom left and top right corners of the segmented
                        image will be considered, with a square window of size
                        given by the --percentage-bc argument, and every
                        segment identified will be set as background. This
                        argument is mutually exclusive with --borders. If
                        --corners is set to be different from 0000, then
                        --borders must be 0000. (default: 0000)
  --k-const K_CONST     Parameter used by the segmentation algorithm to
                        threshold regions in the image. This value controls
                        the degree to which the difference between two
                        segments must be greater than their internal
                        differences in order for them not to be merged. Lower
                        values result in finer region segmentation, while
                        higher values are better to detect large chunks of
                        tissue. Larger images require higher values. (default:
                        10000)
  --minimum_segmentsize MINIMUM_SEGMENTSIZE
                        Parameter required by the segmentation algorithm.
                        Minimum segment size enforced by post-processing.
                        (default: 10000)
  --percentage-bc PERCENTAGE_BC
                        Integer [0-100] indicating the percentage of the image
                        (width and height) that will be considered as
                        border/corner in order to define the background.
                        (default: 5)
  --sigma SIGMA         Parameter required by the segmentation algorithm. Used
                        to smooth the input image with a Gaussian kernel
                        before segmenting it. (default: 0.5)

Examples: See the documentation at https://pyhist.readthedocs.io/
```

S2.2 Graph-based segmentation parameter tuning

Three parameters are important in the outcome of graph-based segmentation: *--k-const*, *--borders*, and *--corners*. Here, we give an overview on what each parameter does. Visual animations for these explanations are available in PyHIST's online documentation (https://pyhist.readthedocs.io/, section "Parameters").

k-const

The *k* constant is used by the graph segmentation algorithm to set a scale of observation. Setting a high value of k will lead to the algorithm detecting segments of a large size, while lowering it will result in a finer region segmentation. In PyHIST's online documentation, we show an animated example of increasing the value of *--k-const* up to the default of 10000. As the value increases, the tissue regions detected as a single unit are larger. Further details on the specific meaning of this parameter are available in the graph segmentation method paper. We find that the default value of *k* tends to work well for many types of WSIs.

Borders and corners

In the graph-based segmentation tiling method, PyHIST identifies tissue regions and assigns a different color to each one. To perform evaluation for each tile to check if it meets the minimum foreground threshold, PyHIST needs to know what is the background color in the image. Since it is not safe to assume that the background color is the most frequent color in the image (for example, a tissue slice might cover almost all the WSI and thus, that region would be the most frequent color), PyHIST relies on either the borders and corners of the mask image to set the background color.

By default, PyHIST looks at the mask color content in the four edges (left, bottom, right, top). How much of the image border is used to determine the background is defined by the *--percentage-bc* argument. The number of pixels that will compose each border is simply calculated as image width x percentage_bc (left and right borders) and image height x percentage_bc (top and bottom borders). In PyHIST's online documentation, we show an example of increasing the *--percentage-bc* in a graph test mode image. As the value increases, PyHIST uses a larger fraction of the image to evaluate the background color. The border sides that PyHIST will use to determine the background color are defined by the *--borders* flag. By default, PyHIST will use all the four borders to determine the background color, however, in some situations it might be desirable to use only

a subset of the borders to determine the background color: for example, in a situation where there are image artifacts present in the borders (due to suboptimal tissue fixing on the slide, etc.).

An alternative way is available in PyHIST to define the background, using the image corners to evaluate the background color in the mask image instead of the borders. The image corners (top left, bottom left, bottom right, top right) to use are controlled through the *--corners* flag. The size of each corner will be calculated as (image width x percentage-bc, image-height x percentage-bc). Only one of the two methods can be used at once: if the *--corners* flag is different from 0000, then the *--borders* flag must set to 0000 and vice versa.
