# Supplementary material for: PyHIST: A Histological Image Segmentation Tool
Source: PLoS Comput Biol. 2020 Oct 19;16(10):e1008349. doi: 10.1371/journal.pcbi.1008349 (PMC7647117; doi:10.1371/journal.pcbi.1008349)
Supplement: S3 Text — Description of data preprocessing, model training and analysis for the TCGA tissue classification use case. (PDF) [file pcbi.1008349.s003.pdf]

Supplementary Text

Section S3: TCGA tissue classification use case

An example use case was developed to show how histological image tiles generated by PyHIST can be used in a ML application. Here, we fit a tissue classifier deep learning model with WSIs from The Cancer Genome Atlas (TCGA) [4]: the goal is to classify image tiles into their tissue of origin based on the histological features present in the tissues in the context of cancer. We employ a total of 36 WSIs, six from each of the following cancer-affected tissues: brain (glioblastoma: GB), breast (infiltrating ductal carcinoma: DC), colon (adenocarcinoma: AC), liver (hepatocellular carcinoma: HC), kidney (clear cell carcinoma: CC), and skin (malignant melanoma: MM). The primary diagnoses for these samples were established by TCGA. In an attempt to avoid possible artifacts during training time, we ensure that: i) all the WSIs within a tissue have the same primary diagnosis, ii) the tissues were obtained from the same sampling sites, iii) samples are sourced from different individuals and, iv) the sample aliquots were destined to the same sequencing/characterization center. Reproducible notebooks for this use case can be found at: https://pyhist.readthedocs.io/en/latest/testcase/

S3.1. Data preprocessing with PyHIST

The slides are downloaded using the Genomic Data Commons' data transfer tool (https://gdc.cancer.gov/access-data/gdc-data-transfer-tool). Using PyHIST, we downsample the original WSIs by a factor of 4x, and require that a tile is composed of at least 40% of tissue content in order to consider it for further analysis. From the 36 WSIs, a total of 7163 tiles with dimensions 512x512 were produced.

We aimed to get 30% of the tiles from each tissue for the test set, and the remaining 70% for the training set. However, in order to avoid data leakage, when performing the tile splitting we should ensure that all the tiles from a single slide go to either the training or the test set. Therefore, from the 6 slides of each tissue, we will choose the slide with the closest number of tiles to 30% of the total tiles to be in the test set, while the remaining five slides will be left for training the model. With this strategy, 5274 tiles (≈74%) were selected for training and 1889 (≈26%) for testing, with the class distribution shown in S1 Table. Class imbalances are fixed at the moment of training the model through data augmentation (the procedure is detailed in section S3.2).

S3.2. Model training

The CNN model to classify the tiles into their tissue of origin (Fig 2a) is fitted with transfer learning from a ResNet152 [5] deep learning architecture pretrained on Imagenet [6]. The model fit was performed with PyTorch (v. 1.6.0+cu101) and torchvision (v. 0.7.0+cu101).

Data augmentation is performed over the training dataset with the following set of ordered transformations for each tile: a randomly resized crop (from 80% of the original size up to 100%, where no crop is performed) is taken from the 512x512 input tile, a random rotation of in the range of (-15, 15) degrees is performed, the tile can be flipped horizontally with probability 0.5, the tile is then resized to 224x224 (which is the default size for ResNet152), then transformed into a [0, 1]-normalized tensor and finally each pixel is normalized across the color channels using ImageNet's mean ([0.485, 0.456, 0.406]) and standard deviation ([0.229, 0.224, 0.225]). For the test set, only the last three steps of the transformations are applied (i.e. tiles are resized to 224x224, [0, 1]-normalized tensor, and ImageNet color-normalized). The sets of transformations vary for each training epoch.

In order to fix class imbalances in the training set, within each training epoch: i) the order of the training data is randomly shuffled, and ii) the training batches are created using weighted random sampling: tiles are selected using a weight of $1/n_{class}$, where n_{class} is the number of tiles in the training set for the class of a given tile. With this training scheme, we guarantee that the class representation within a training batch is fair (S6 Fig). Since randomness is involved in performing several data augmentation steps, whenever a tile is selected a different set of transformations will be applied over it, effectively being able to balance the data classes using the original data as the source.

The fully connected layer at the end of the ResNet152 model is replaced by a sequential container with the following modules: linear layer mapping to 1000 features, ReLU, dropout with a probability of 0.4, linear layer mapping to the 6 features (corresponding to the classes), and finally LogSoftmax transformation is applied over these. Negative log-likelihood loss is used to perform the optimization with the Adam algorithm. The optimizer's default learning rate is used, with a step decay of 0.1 (multiplicative factor) each two epochs. The model is fit using a single Tesla T4 GPU, using a batch size of 64.

First, we freeze (i.e. we do not require gradients) all the parameters of the model, except for the parameters in the sequential container described above, and train the model for 6 epochs. We then unfreeze all the parameters in the model and train it for 10 more epochs, reaching 95% accuracy over the test set. The confusion matrix for the predictions in the test set is shown in S2 Table.

Once the model is trained, we perform inference over all the tiles to obtain the feature vector (of size 1x1000 per tile) generated at the first linear layer in the sequential container described above. We stack these vectors to generate a matrix of size 7163 x 1000, which will be used to perform dimensionality reduction and clustering.

S3.3 Dimensionality reduction and clustering

Using the feature vector matrix generated at the end of the model training notebook, we perform dimensionality reduction using t-SNE (Fig 2b), and observe 6 clusters of observations that correspond to the tissue class labels. The clusters are well separated, except for the case of skin (MM) and breast (DC) clusters where a small overlap of tiles can be observed: these tiles correspond to those that were classified incorrectly by the model (see S2 Table). Hierarchical agglomerative clustering with complete linkage is performed over Pearson's correlation matrix of the feature vectors (S7 Fig). High within-tissue correlation blocks are observed, suggesting that the generated feature vectors encode well the tissue morphology (as expected, since most of these tissues have quite distinct histological features within the context of cancer).

S3.4 Tile assessment

We performed a visual inspection of the five tiles predicted with the highest probability for each tissue and cancer type (Fig 2a) in order to corroborate that the characteristics observed in the tiles are compatible with the histopathological diagnosis generated by TCGA at the slide level:

1. First row: The images of the row seem equivalent. They show highly anaplastic and pleomorphic cells. Multinuclear cells and vascular proliferation can be identified. The images are compatible with glioblastoma.
2. Second row: The images of the row seem equivalent. Desmoplastic stroma is apparent. Cords of poorly differentiated cells can be observed in-between the stroma's bundles. Clear invasion of the stroma can be observed in several points. Tubular structures were not identified. The images are compatible with infiltrating ductal breast carcinoma.

3. Third row: The images of the row seem equivalent. Well-differentiated glands secreting mucin can be observed. The epithelium is composed of pseudostratified columnar cells and goblet cells. Stroma between the glands is desmoplastic. The images are compatible with colon adenocarcinoma.
4. Fourth row: The images of the row seem equivalent. Clear cytoplasm cells with small nuclei were observed. They are disposed in nests and cords. A rich vasculature network is observed. The images are compatible with clear cell renal carcinoma.
5. Fifth row: Histopathologic features of images of the row seem equivalent. They show highly eosinophilic cells resembling hepatocytes. The cell arrangement seems trabecular with occasional lobular like structures without central veins. Cells are pleomorphic and contain lipid droplets in the cytoplasm. Some Mallory bodies can be identified. The images are compatible with hepatocellular carcinoma.
6. Sixth row: Images of this row seem equivalent. They show atypical epithelioid-like melanocytes in the epidermis and dermis. Cells show marked nuclear pleomorphism and intense eosinophilia of the cytoplasm. The images are compatible with malignant melanoma.

References

1. Canny J. A computational approach to edge detection. Readings in computer vision. Elsevier; 1987. pp. 184–203. doi:10.1016/B978-0-08-051581-6.50024-6
2. Felzenszwalb PF, Huttenlocher DP. Efficient Graph-Based Image Segmentation. Int J Comput Vis. 2004;59: 167–181. doi:10.1023/B:VISI.0000022288.19776.77
3. Aguet F, Barbeira AN, Bonazzola R, Brown A, Castel SE, Jo B, et al. The GTEx Consortium atlas of genetic regulatory effects across human tissues. Science 2020;369: 1318-1330. doi:10.1126/science.aaz1776.
4. Cancer Genome Atlas Research Network, Weinstein JN, Collisson EA, Mills GB, Shaw KRM, Ozenberger BA, et al. The Cancer Genome Atlas Pan-Cancer analysis project. Nat Genet. 2013;45: 1113–1120. doi:10.1038/ng.2764
5. He K, Zhang X, Ren S, Sun J. Deep residual learning for image recognition. IEEE Conference on Computer Vision and Pattern Recognition (CVPR). IEEE; 2016. pp. 770–778. doi:10.1109/CVPR.2016.90
6. Deng J, Dong W, Socher R, Li L-J, Li K, Fei-Fei L. ImageNet: A large-scale hierarchical image database. 2009 IEEE Conference on Computer Vision and Pattern Recognition. IEEE; 2009. pp. 248–255. doi:10.1109/CVPR.2009.5206848
