# Supplementary figures and images for: PyHIST: A Histological Image Segmentation Tool
Source: PLoS Comput Biol. 2020 Oct 19;16(10):e1008349. doi: 10.1371/journal.pcbi.1008349 (PMC7647117; doi:10.1371/journal.pcbi.1008349)

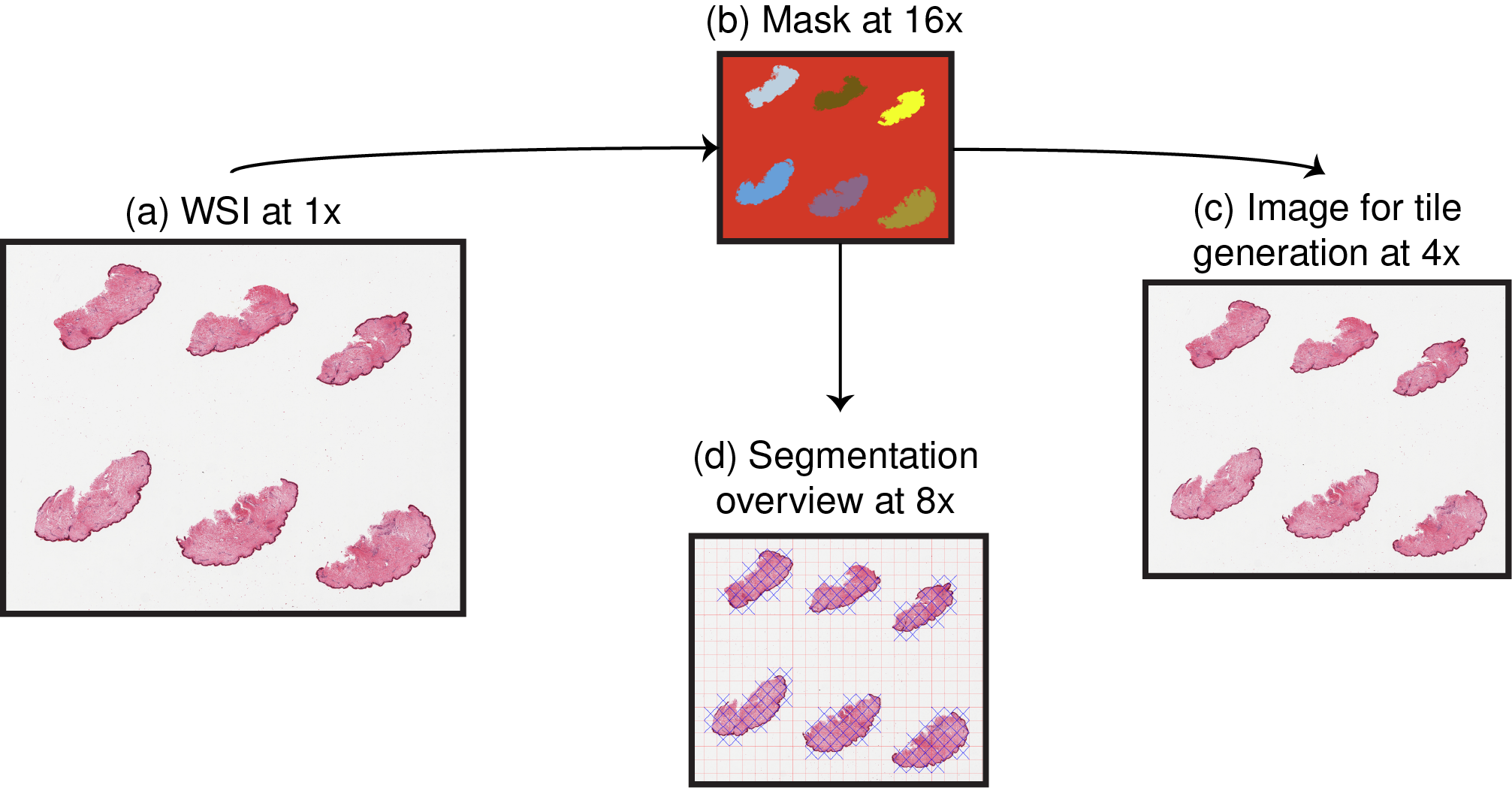

Supplement: S1 Fig — (a) WSI at its original resolution (1x). (b) The mask can be generated and processed at a given downsampling factor. A smaller resolution will lead to a faster segmentation. (c) The output can be requested at a given downsampling factor. (d) The segmentation overview image can also be generated at a given downsampling factor. The dimensions in all steps are matched to ensure that the tile sizes and grid are consistent. The downsampling choices for all the steps are independent of each other. (PNG) [file pcbi.1008349.s004.png]

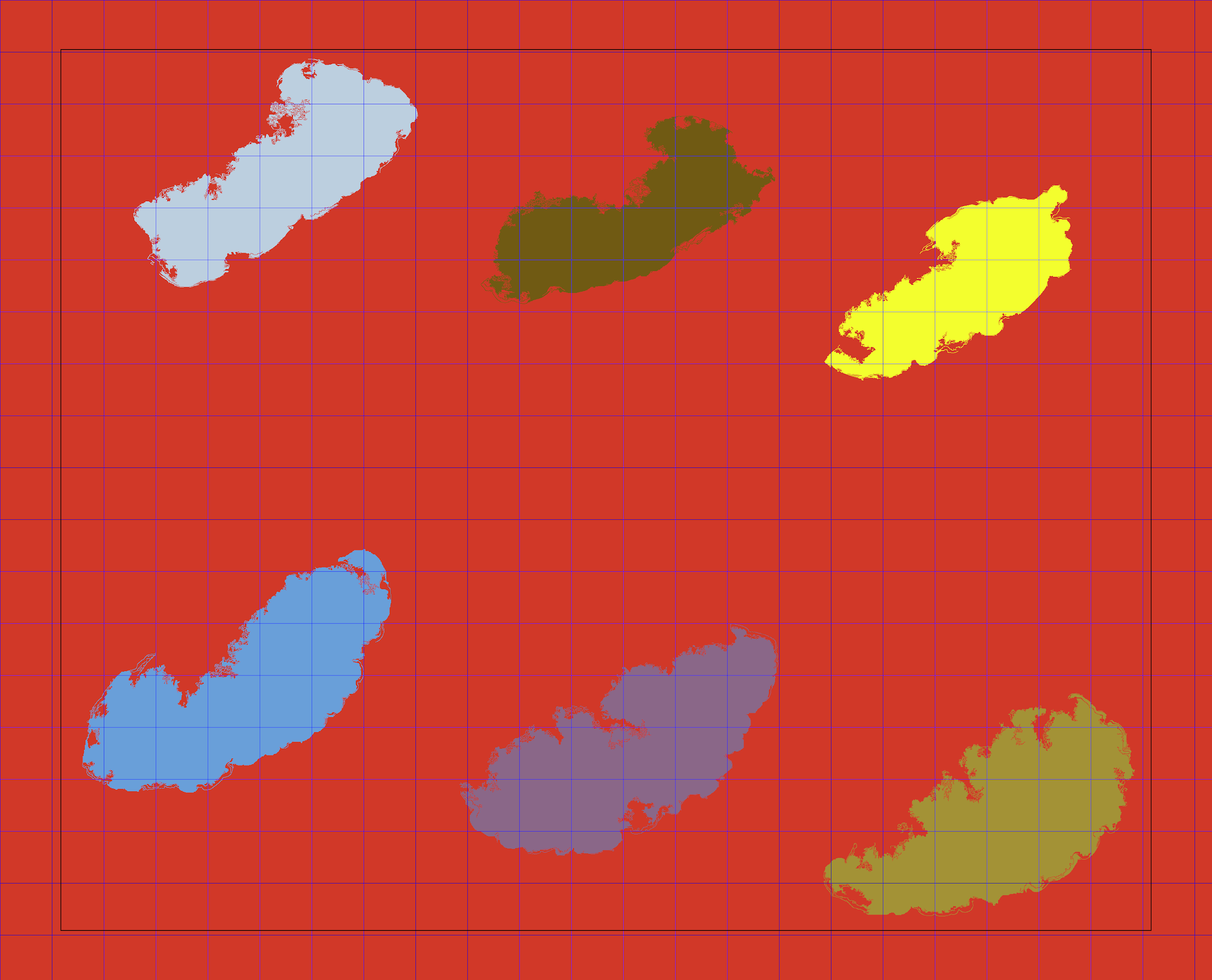

Supplement: S2 Fig — Test mode allows the user to see how the image mask will be with the chosen segmentation parameters and tile dimension configuration, before proceeding to generate the individual tile files. The black border defines the region of exclusion for tissue content placed within the edges of the slide (see—borders and—corners arguments, and section 2.2 in S2 Text). (PNG) [file pcbi.1008349.s005.png]

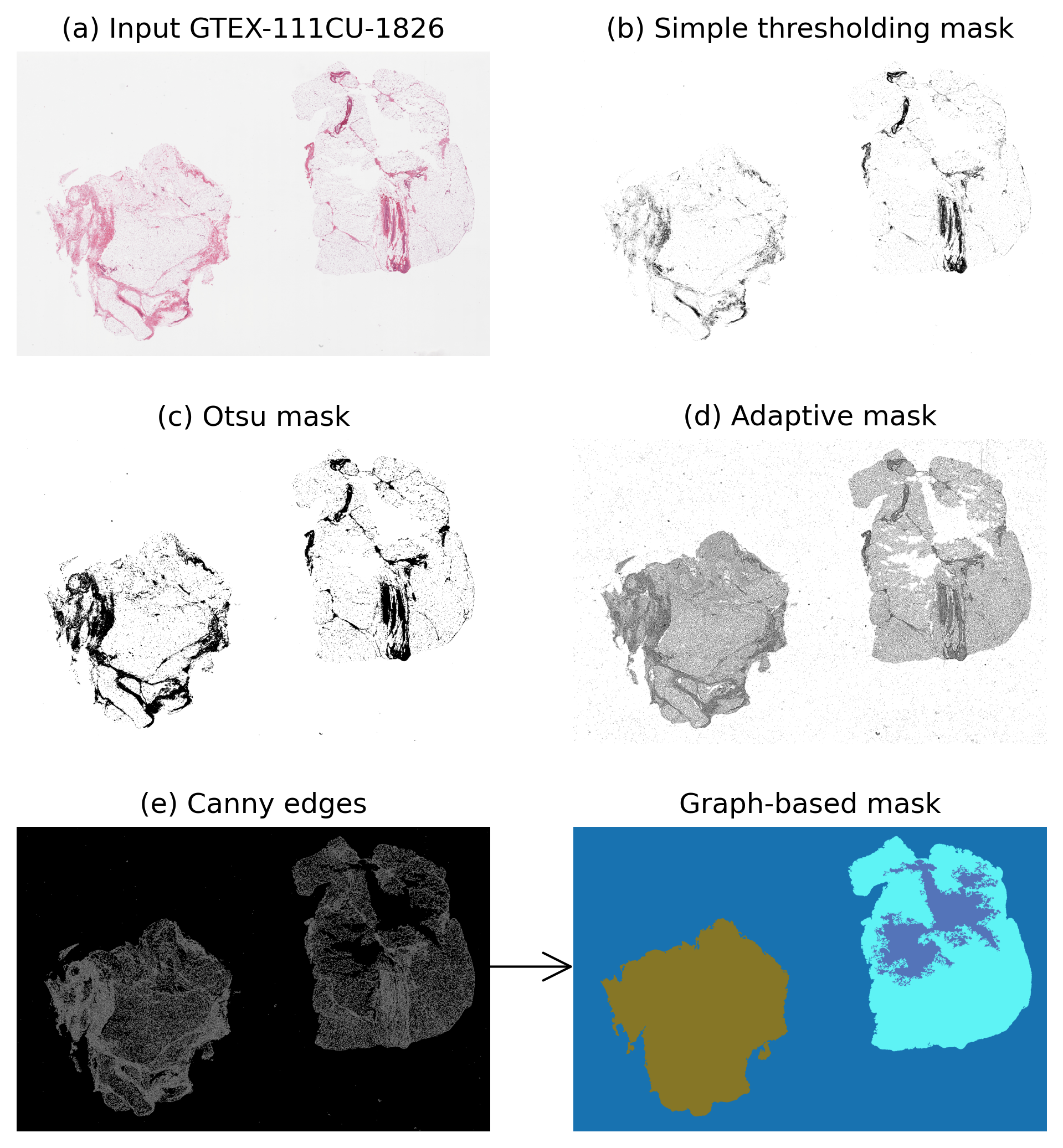

Supplement: S3 Fig — (a) Adipose tissue WSI from the GTEx project, from sample GTEX-111CU-1826. Thresholding-based masks (b-d) are generated by first converting (a) into grayscale and then applying the corresponding thresholding method. Note that simple thresholding is shown here for completeness but only Otsu and adaptive are implemented in PyHIST due to their overall better performance when compared to simple thresholding. In the graph-based method, an image with highlighted edges is first generated through a Canny edge detector (e, left) and then the connected components are labeled through graph-based segmentation (e, right). (PNG) [file pcbi.1008349.s006.png]

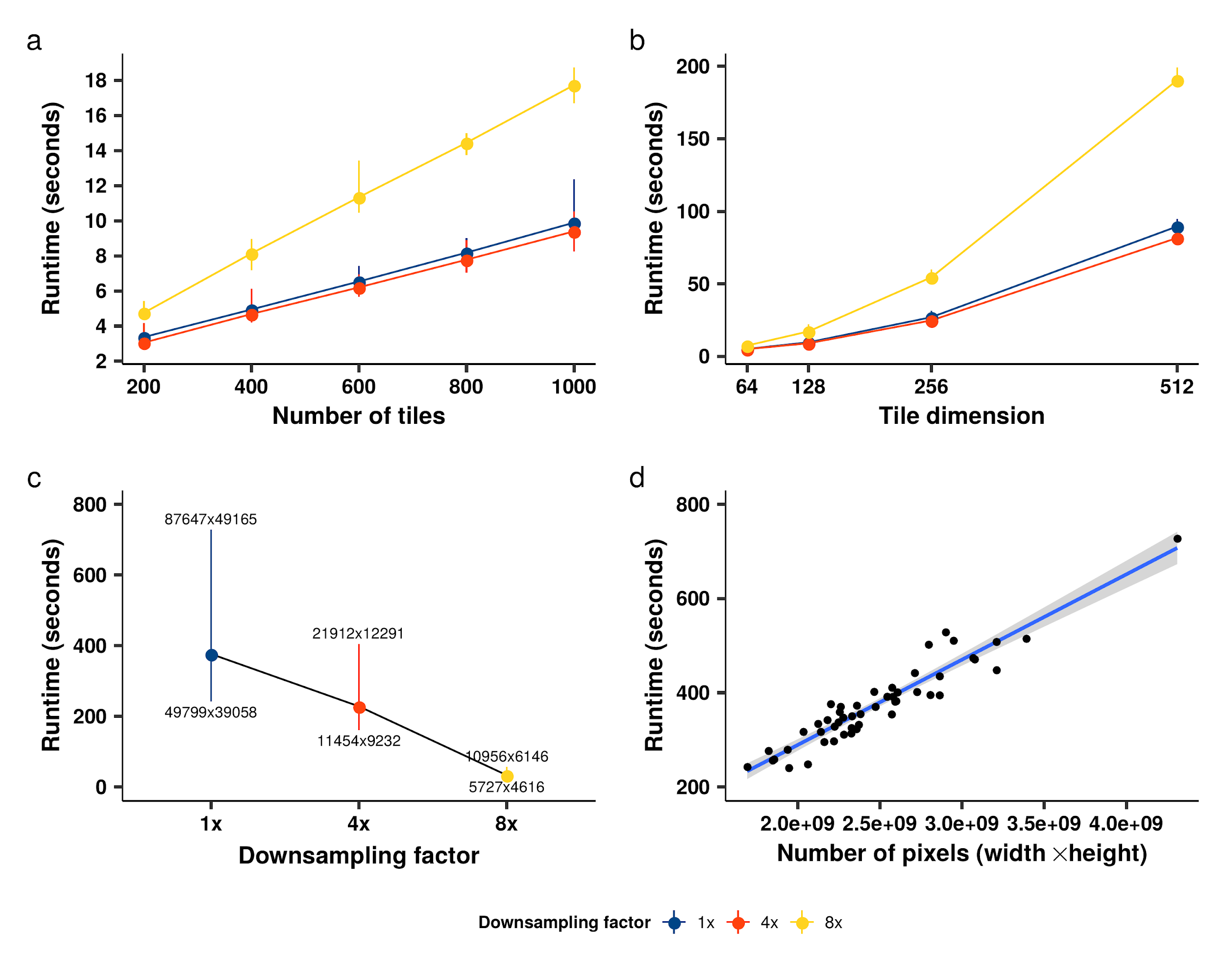

Supplement: S4 Fig — (a) Execution time to perform random sampling (y-axis) of a varying number of tiles (x-axis) at different downsampling factors for the WSI shown in S1 Fig. For each combination of number of tiles and downsampling factor, the sampling was repeated 30 times. Each dot represents the average running time across the 30 runs, while the interval shows the range between the maximal and minimal running time. (b) Execution time to perform random sampling of 1000 tiles (y-axis) at different tile dimensions (x-axis) at different downsampling factors for the same WSI in (a). Each combination was repeated 50 times, with each dot showing the average runtime. (c) Segmentation runtime of 50 Stomach WSIs from the GTEx project, at different downsampling factors, at a tile size of 256x256. Each dot represents the average execution time. Each interval shows the range between the fastest and slowest segmentations, while the labels show the dimensions of the corresponding WSIs. (d) Segmentation runtime (y-axis) at 1x resolution for the 50 Stomach WSIs, with respect to the number of pixels in the WSI (x-axis). (PNG) [file pcbi.1008349.s007.png]

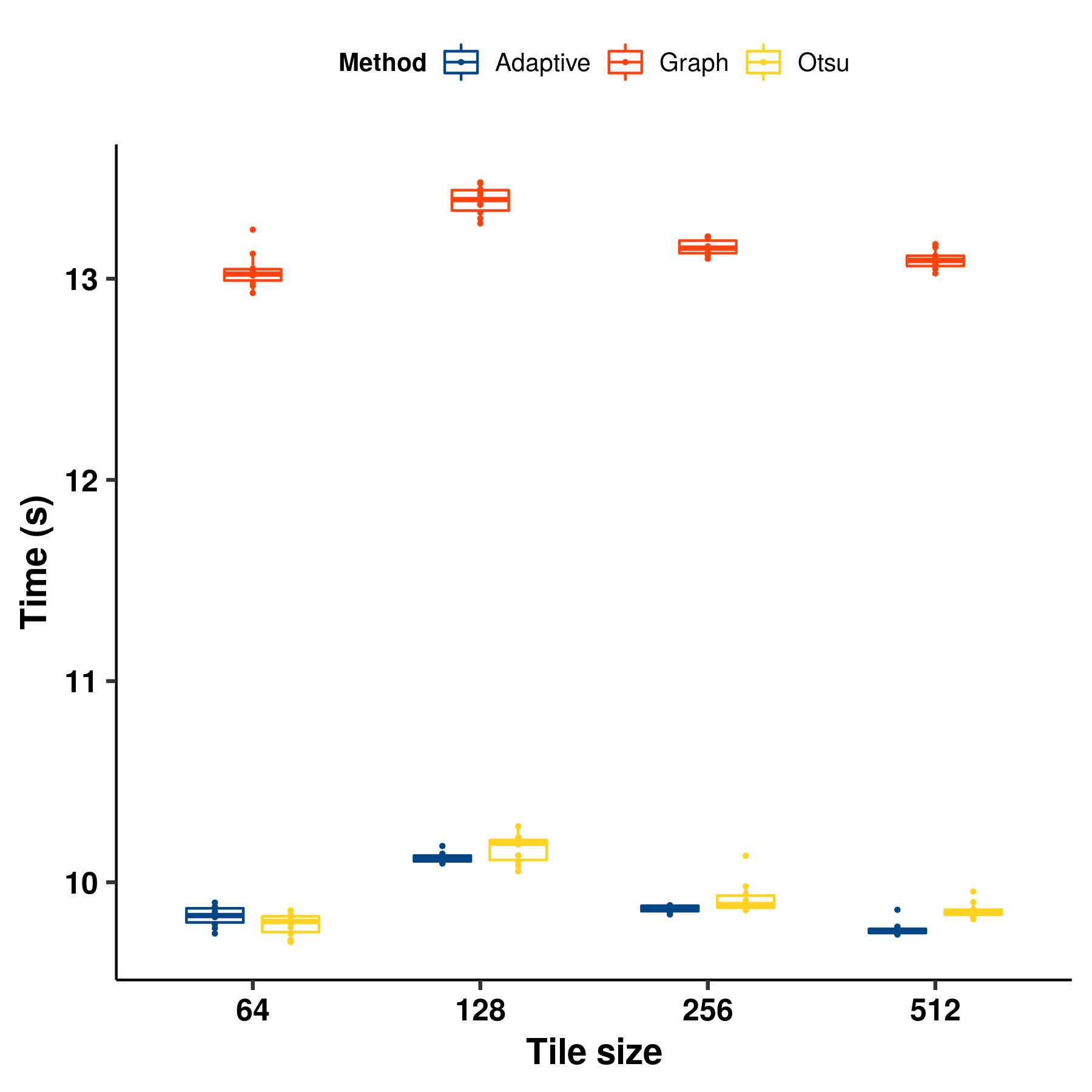

Supplement: S5 Fig — Tile extraction was evaluated for the three different methods at four different settings of tile size. Each method + tile size combination was repeated ten times to show runtime variability. (PNG) [file pcbi.1008349.s008.png]

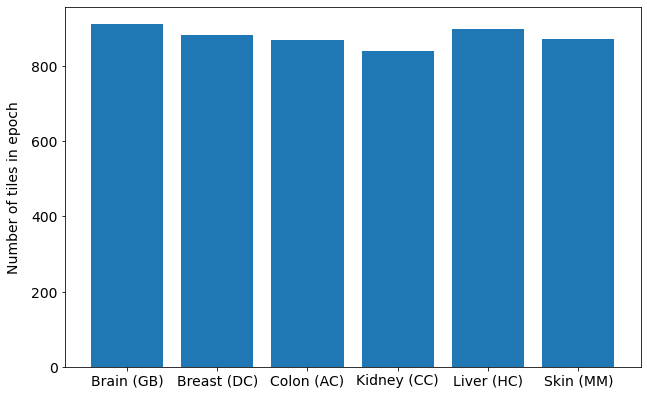

Supplement: S6 Fig — Within each training epoch, weighted random sampling is performed to create batches with a fair distribution of tiles among the classes. Even if the sample sizes in the training dataset are different among the classes, the balance in the number of tiles per epoch is obtained through data augmentation. (PNG) [file pcbi.1008349.s009.png]

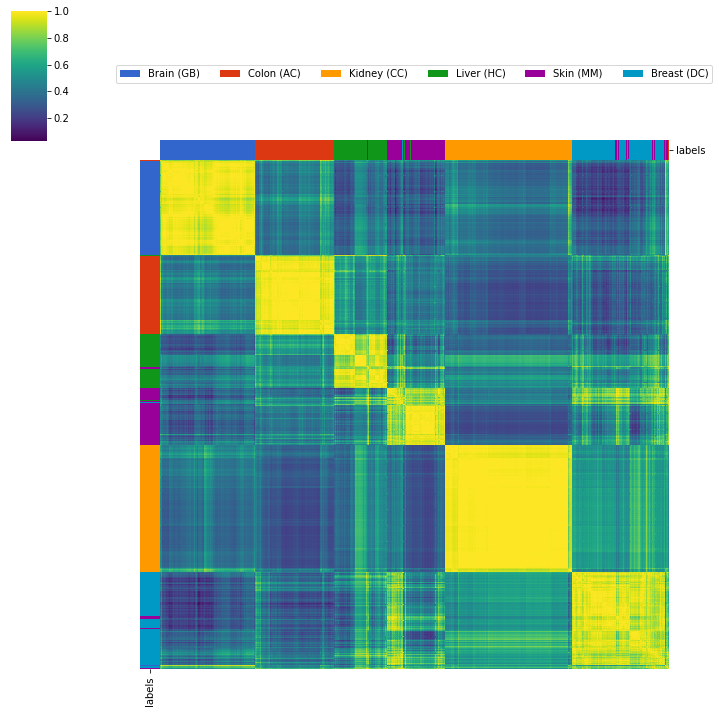

Supplement: S7 Fig — Heatmap of Pearson’s correlation matrix between the feature vectors obtained for each TCGA tile. Rows and columns are reordered with hierarchical agglomerative clustering. (PNG) [file pcbi.1008349.s010.png]

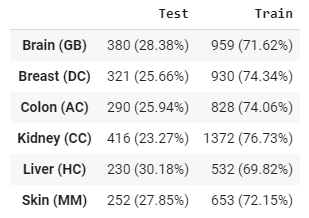

Supplement: S1 Table — (PNG) [file pcbi.1008349.s011.png]

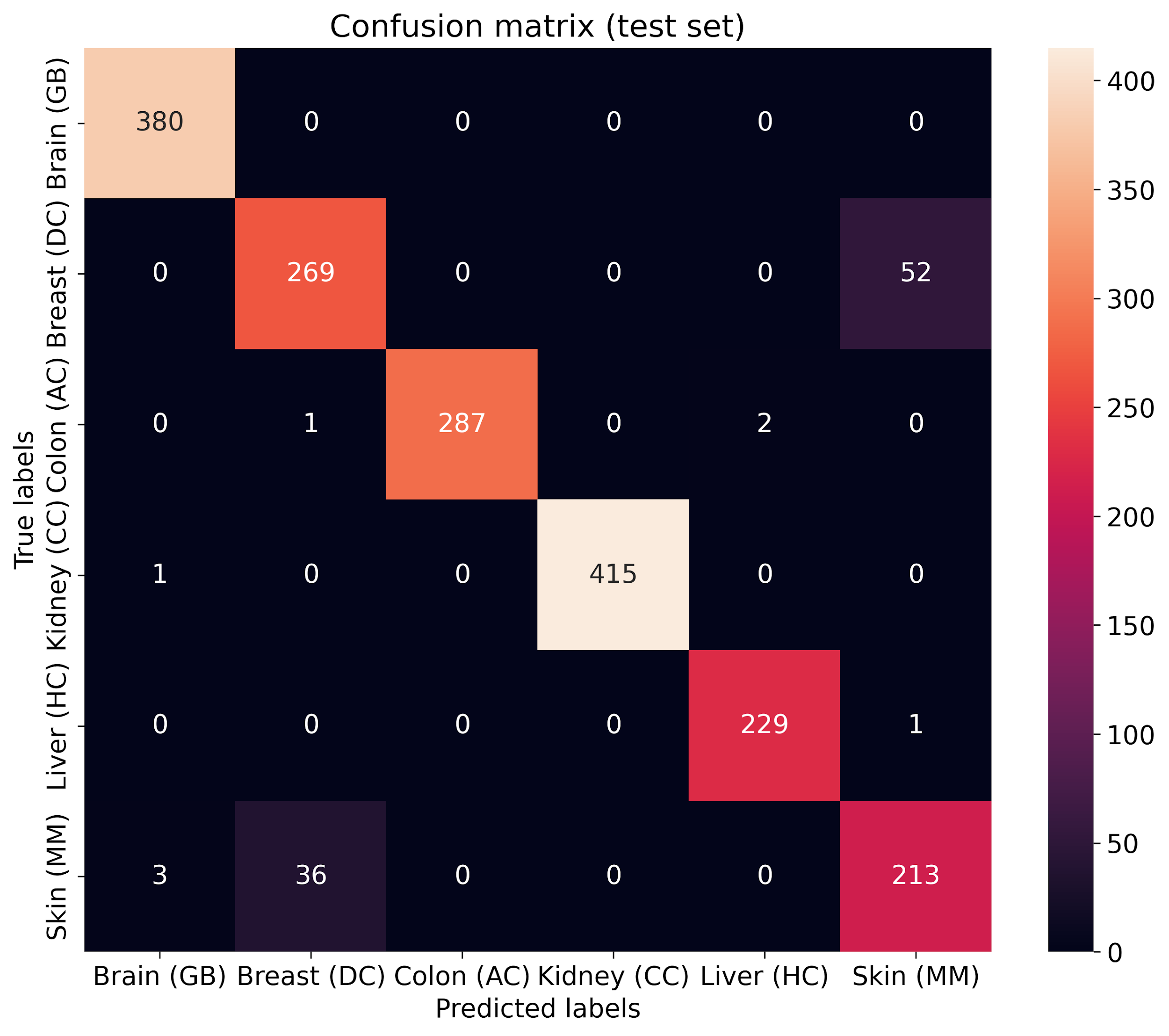

Supplement: S2 Table — (PNG) [file pcbi.1008349.s012.png]
