# Supplementary material for: PyHIST: A Histological Image Segmentation Tool
Source: PLoS Comput Biol. 2020 Oct 19;16(10):e1008349. doi: 10.1371/journal.pcbi.1008349 (PMC7647117; doi:10.1371/journal.pcbi.1008349)
Supplement: Attachment — Submitted filename: Reviewers_comments_R1.pdf [file pcbi.1008349.s013.pdf]

Reviewer #1

R.1.1 The paper introduces a simple and easy to use open-source tool for slide tiling, which is useful for histopathology image analysis. I have to acknowledge there are currently inadequate tools available that make tiling of digital slides simple, and I had to write my own scripts to tile my slides when analyzing whole slide images. Overall, my experience with the application was positive; I was able to create tiles from svs and TIFF files in a short amount of time, with a short learning curve.
We thank the reviewer for the comments and are glad that the experience using the tool was positive. PyHIST was developed when the need to perform tiling of digital slides arose in our own projects, and we hope that it can also be of use for computational biologists working with histological images.

R.1.2. The setup and installation was relatively easy; the Docker version of the software worked without problems on Ubuntu and Windows 10 based machines. A few problems were encountered when the program was installed through Anaconda using the program's accompanying installation instructions: in the Ubuntu machine, 'cv2' was reported to be missing; in Windows 10, several libraries were missing. I was able to get the program running in Ubuntu and Anaconda after manually installing OpenCV. I did not attempt to manually fix the Anaconda installation in Windows due to the large number of missing libraries. I am not sure if this is due to the unique setup of my computer, or the program was not tested with Windows 10 and Anaconda.
We thank the reviewer for spotting these issues. With respect to:
1) Missing "cv2" library in Ubuntu: We were able to reproduce this. The conda environment file has now been fixed. We have also tested the conda environment installation inside an independent Docker container with only base Ubuntu packages installed, and PyHIST now executes without dependency issues. To prevent future issues with dependencies, we have also added continuous integration (Travis) to the code repository to test the software on each change.

2) Anaconda and Windows 10: PyHIST depends on Openslide to process the histological images, and this library is currently not available through conda in Windows 10. Standalone installation of Openslide in Windows 10 requires i) installing Visual C++ build tools and ii) modifying a system variable. In addition, the image segmentation subroutine used in PyHIST needs to be compiled using external tools. Since setting up the previous might not be straightforward for many end-users, we amended the installation guide to encourage Windows 10 users to use the Docker image which already contains all the necessary dependencies, and clarified that the conda environment is currently compatible with Linux/macOS.

Suggested Correction:

R.1.3. Lines 22 - 23 states “Histopathological images are routinely used in the diagnosis of many diseases, notably cancer.” This can be misinterpreted as saying that pathologists make their diagnoses predominantly through whole slide images (WSIs). Although WSI is becoming more widespread in pathology departments, most pathologists still render their diagnoses by examining glass slides under a microscope. This statement has to be corrected/modified to reflect that whole slide images are still not being used by majority of pathologists to sign out their cases, although there is an increasing adoption of whole slide scanning technologies in pathology departments.
We agree with this, and have clarified this statement to avoid misinterpretation: "Currently, these glass slides of fixed tissue samples are the preferred method in pathology laboratories around the world to make clinical diagnoses [2], notably in cancer [3]. However, the increasing automation of WSI acquisition has led to the development of computational methods to process the images with the goal of helping clinicians and pathologists in diagnosis and disease classification [4]."

Future direction:

R.1.4. There is more potential in this software, which can accommodate additional features in the future while retaining its simplicity. Aside from adding new features, I believe adding a Graphical user interface (GUI) version of the program would increase the application’s user base, and be helpful for those who are less computer savvy and have no experience in using the command line.
This is a good suggestion. Although we have originally developed PyHIST with a specific user base in mind, we agree that having a GUI can certainly be useful and ease the learning curve for those who are new to the field of image processing for machine learning applications. To this end, we have now refactored PyHIST’s source code to easily incorporate these extensions in the future, as well as integrating new features (thresholding methods for mask generation, experimental support for additional file formats, cleaner execution logs) with respect to the initial submission.

We have elaborated on this in the future direction section of the text: “Future directions of improvement include adding support for more histological image formats and features to save tiles into specialized data structures, as well as the inclusion of a graphical interface to ease the learning curve for users who are new to the field of image processing for ML analyses.”

Reviewer #2

R.2.1: The manuscript submitted by Muñoz-Aguirre and colleagues aims to describe the development of PyHIST which is a histological image segmentation tool. Overall, this manuscript presents results that would be of interest to the community of scientists and computational biologists concerned with this problem. However, there are major issues in this manuscript that prevent us from recommending that this manuscript be accepted in its current state.
We thank the reviewer for the thorough evaluation of our manuscript and constructive feedback provided. We have given careful consideration to the raised points which have helped to significantly improve both PyHIST and the manuscript relative to the original submission.

Major:

R.2.2: 1) Abstract: highlights that preprocessing enabled by PyHIST involves image scaling, segmentation, and eventually tile extraction to clearly mention the utility of PyHIST.
In this revision, we have included new methods to perform tile extraction, and have thus amended this part of the abstract to give a clear idea of PyHIST's intended usage: "[...] an easy-to-use, open source whole slide histological image tissue segmentation and preprocessing command-line tool aimed at tile generation for machine learning applications. From a given input image, the PyHIST pipeline i) optionally rescales the image to a different resolution, ii) produces a mask for the input image which separates the background from the tissue, and iii) generates individual image tiles with tissue content."

R.2.3: 2) Introduction: The paper correctly addresses the need for standardization of the tiling and patch-creating pipeline for researchers working in this area to prevent dataset-specific biases. Although, as far as saving research time is concerned, currently, WSI preprocessing requires developing custom scripts, but once a process is established researchers can typically use similar code for subsequent tiling for all projects. Therefore, PyHIST may only save a significant amount of time at the initial phase.
The reviewer is correct. We have experienced in our own projects that the data preparation stage can be significantly time-consuming, and thus, developed PyHIST precisely with the goal of helping users save time during this phase. However, as the reviewer also points out, PyHIST also has the added value of standardizing across studies the preprocessing of whole slide histological images. We now explicitly state this in the text: "To systematize the WSI preprocessing procedure for these applications, and in order to streamline the data preparation stage at the initial phase of a ML project by avoiding the need of creating custom image preprocessing scripts, we developed PyHIST, a command-line based pipeline [...]".

R2.4: 3) Facts have been mentioned without references – we have mentioned a few examples but urge the authors to add extensive references:

- - lines 22 (citation for WSI obtaining process required),
- - 23 (citation for use in cancer),
- - 25 (citation to support the claim of development of computational methods for disease diagnosis and classification), and
- - 33 (cite literature to support histopathological images capturing endophenotypes that provide crucial information when correlated with molecular and cellular data).
- - In a similar way, kindly provide references at lines 37, 46, 50

The main text has been revised to include these references. We have also ensured that, to the best of our knowledge, the relevant literature is also cited throughout the rest of the text.

4) Design and Implementation:

R.2.5: - It's not clear why the authors are interested in highlighting edges within tissue fragments rather than outlining the entire fragment. Figure 1b resembles a grayscaled WSI. A similar result as Figure 1b can be reached with less computation by just binarizing the WSI using a threshold to separate background from foreground. Does edge detection provide any unique benefits over binarizing the WSI?
Although in several cases the use of a thresholding technique for binary mask generation can be sufficient to select the slide tiles with tissue content, we found that it might not perform well in cases where the differences between the background and tissue are more subtle. We have encountered examples of such cases in our research projects when dealing, for example, with WSIs (H&E staining) of adipose or breast tissues.

Here, we show an example of such a case with a H&E-stained adipose tissue WSI from the GTEx project (Fig. R1a). We compared the masks generated by different methods: simple cutoff thresholding (Fig. R1b), Otsu's thresholding (Fig. R1c), adaptive thresholding (Fig. R1d), and graph-based segmentation (Fig. R1e). In the graph-based method, an image highlighting the tissue edges is generated first through a Canny edge detector (Fig. R1e, first panel), which is then processed by the graph-based segmentation method, leading to a clean mask with closed objects (Fig. R1e, second panel).

In PyHIST, the mask is used to select the tiles that meet a certain tissue content (foreground) threshold. Thresholding methods (Fig. R1b-d) might not capture all the tissue details, and thus, it is not straightforward to generate closed objects in tissues with a composition like that of adipose: further morphological operations would be needed to generate a mask like the one generated by the graph-based method (we elaborate more on this in the reviewer's comment R.2.6.). As a consequence, thresholding methods might not perform as well in these types of scenarios.

We initially decided to choose the graph-based segmentation method since it covers the largest number of use cases by being configurable in its parameters to suit different tasks: for example, not all users might be interested in keeping tiles with little foreground content. Having said this, the reviewer's point is well taken: in scenarios where the tissues have a homogeneous composition, the use of thresholding methods can simplify the pipeline usage. Thus, we have now implemented two additional alternatives in PyHIST to generate the input image mask: Otsu and adaptive thresholding. The tile generation method can now be easily selected by the user at runtime. We have also now included these mask comparisons in Supplementary Text S1 and Supplementary Figure S3.

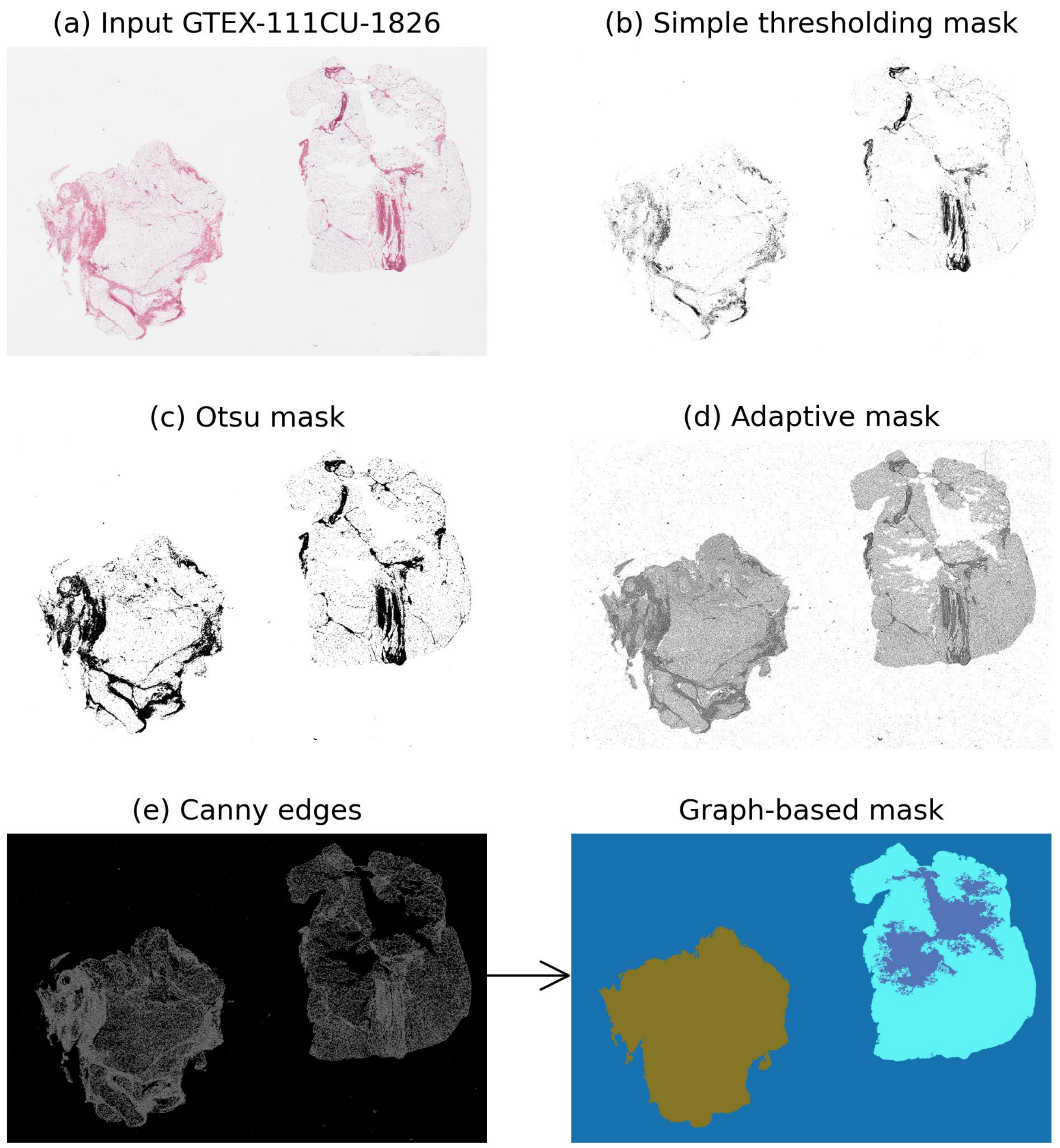

Fig. R1: Method comparison for mask generation. (a) Adipose tissue WSI from the GTEx project. The next three panels show masks generated by thresholding methods: (b) simple thresholding, (c) Otsu's thresholding and (d) adaptive thresholding. In panel (e), the first image shows the edges detected by the Canny edge detector and the second image shows the mask generated when applying the graph-based segmentation method over the previous image.

R.2.6: The graph-based segmentation algorithm can perform unsupervised segmentation on complex images, but in this case the algorithm just needs to detect the connected objects. If the input image is a binary mask (foreground and background), there are many simple functions to label contiguous/interconnected objects and produce an output similar to Figure 1c. Is graph-based segmentation used because it works well with edge detection inputs? How does it compare computationally to other connected-component labeling techniques such as Python Skimage's measure.label function?
This is correct - graph segmentation tends to work well together with images generated by the edge detector. Detecting connected components (for example, with the use of the scikit-image's measure.label function) over the binary masks generated by thresholding might not work well on cases like the one described on R2.5 since the tissue slices cannot be easily detected as closed objects.

Here, we take the left tissue segment of Fig. R1a to demonstrate (Fig. R2a). First, we generate the Otsu and adaptive thresholding binary masks (Fig. R2b-1 and R2c-1). If we apply measure.label directly over these, more than a thousand individual regions are generated. Since the masks generated through these thresholding methods are non-closed objects with "peppered" noise, they might benefit from further processing (morphological operations, for example) with the goal of generating a mask similar to the one generated by the graph-based segmentation. We show examples of performing such an operation to try to clean and close these regions (Fig. R2b-2 and R2c-2) and find that the final masks generated through scikit's label method (R2b-3 and R2c-3) are missing details and the detected regions are very granular. Finding a combination of morphological operations that are robust enough to work with these complex types of WSIs is not trivial.

As mentioned before, the level of detail that should be kept will depend on the end-user's specific task. This can be controlled through the graph-based segmentation parameters. Otsu/adaptive thresholding, which are also now available as methods in PyHIST, will simply ignore regions that can be easily mistaken as background. With respect to runtimes - Otsu and adaptive thresholding require less computation than the graph-based method as correctly pointed out by the reviewer on R2.5. We have now added details of runtimes in Supplementary Text S1.

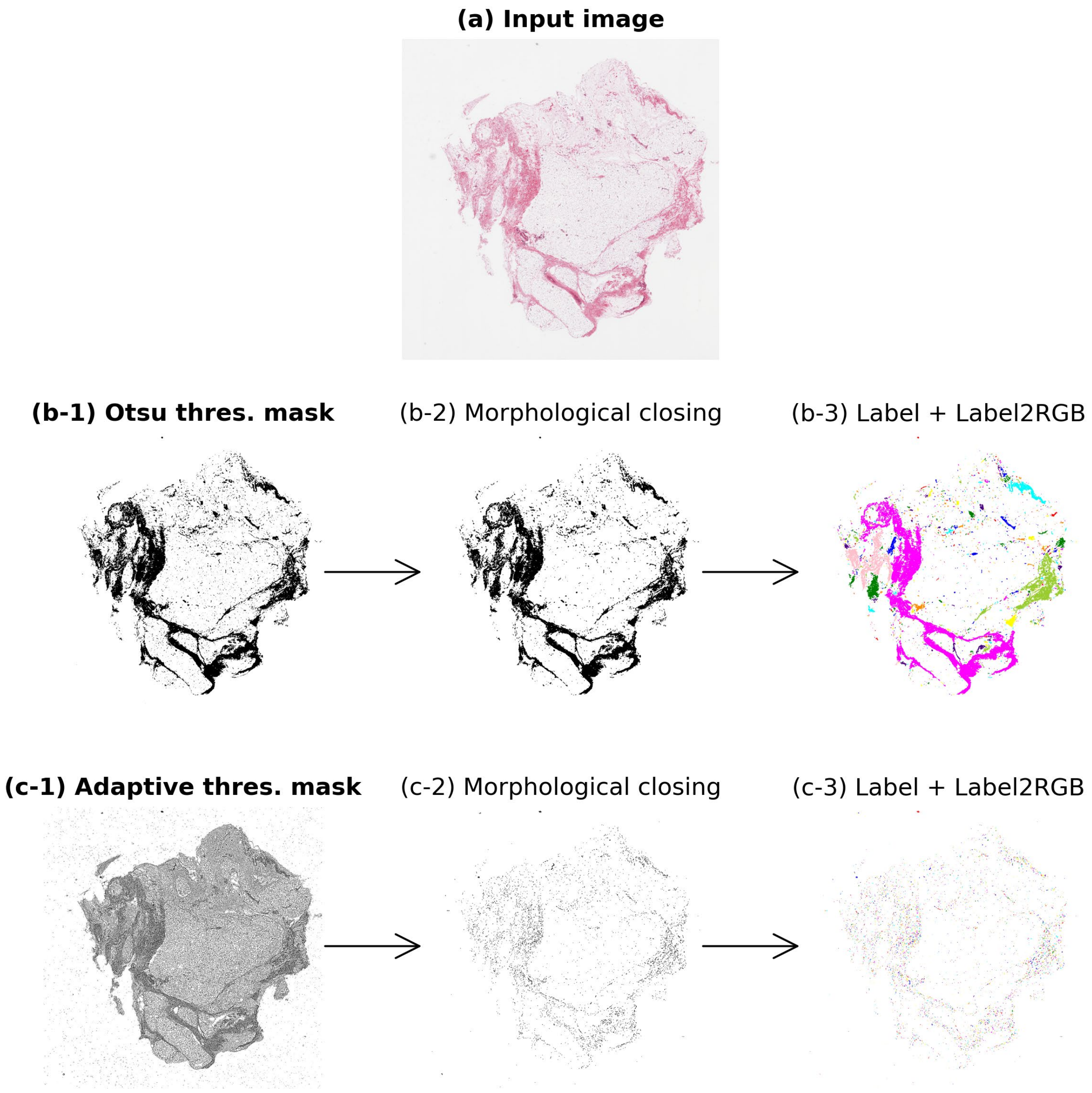

Fig. R2: Connected-component labeling over thresholding masks. (a) A tissue fragment from GTEx adipose tissue WSI GTEX-111CU-1826. (b-1) and (c-1) are the Otsu and adaptive masks generated when thresholding image (a); (b-2) and (c-2) are the result of applying morphological closing over the masks; (b-3) and (c-3) are the labeled components generated by the scikit-image's label method, and color-coded with the label2rgb function.

R.2.7: - Why are steps (b) and (c) needed in the PyHIST pipeline in Figure 1? Red gridlines still appear to tile the entire WSI and then some tiles are not stored based on a background threshold. How are the tissue fragment labels from (c) used?
PyHIST decides which tiles to keep by using the mask shown in Fig. 1c: it evaluates each tile in the mask and uses the tissue fragment labels to assess if the percentage of foreground (area of the label with respect to the total area of the tile) in the tile meets a minimum threshold defined by the *--content-threshold* flag. We have clarified this on the main text:

"The PyHIST pipeline involves three main steps: 1) produce a mask for the input WSI that differentiates the tissue from the background, 2) create a grid of tiles on top of the mask, evaluate each tile to see if it meets the minimum content threshold to be considered as foreground and 3) extract the selected tiles from the input WSI at the requested resolution. By default, PyHIST uses a graph-based segmentation method to produce the mask."

In graph-based segmentation mode, an image that highlights the edges (b) is first created through a Canny edge detector and then segmented, as described in previous answers and in the main text. The image with the red gridlines is produced to aid the user in having an overview of which tiles were selected. The number of tiles that are selected will depend on the value of the *--content-threshold* flag. In PyHIST's online documentation, we show an example of how the number of selected tiles can change based on this threshold.

5) Results:

R.2.8: - Details of the deep learning model have not been provided – patches detected correctly have vague histology that is shown in Figure 2 A (explained below). We suggest a pathologist review of the deep learning model results. Additionally, the connection between a better model accuracy on the dataset and the validity of the pre-processing steps has not been made.
We have now included detailed explanations about the deep learning model in Supplementary Text S3. The top histological tiles have now been reviewed; we elaborate on this in the reviewer's comment R.2.12. We refrained from making statements in terms of the use case classification accuracy due to the reasons mentioned in the response to the reviewer's comment R.2.10. We consider that the clustering and t-SNE plots (Fig. 2b, Supplementary Figure 7) show that the tiles recapitulate the tissue morphology correctly since the expected tissue clusters are observed, and are thus a good validation procedure: if the tiles contained noise or background, a separate cluster would be observed with mixed tiles of all types.

R.2.9: - The partitioning of training and test sets can be the most time-consuming pre-processing steps of the ML process. Tiles from the same WSI should be constrained to the training or test sets. It is difficult to satisfy this constraint, while also managing the percentage of tiles in the test set and class imbalances. This process is not a built-in feature of PyHIST and it is unclear in the paper if PyHIST assists with this aspect of the ML pipeline at all.

We designed PyHIST to aid end-users in the tile extraction procedure for ML applications while at the same time being agnostic to the specific details of that application. For example, a user might be interested in performing only inference over tiles using pre-trained model weights in which case they might not need to perform such a data partition. For this reason, we have not explicitly included functionality in PyHIST to perform these types of data partitions.

In the case of a classification task, however, we concur that by ensuring that tiles from the same WSI are constrained to a single set (either training or testing) helps in reducing data leakage at the moment of obtaining model performance metrics. To this end we have amended the TCGA use case by i) increasing training data by one slide per tissue, ii) performing the training/testing partition at the WSI level, and, iii) performing weighted random sampling per training epoch to guarantee a fair distribution of the classes. This information is now explicitly detailed in the Supplementary Text S3.1:

"We aimed to get 30% of the tiles from each tissue for the test set, and the remaining 70% for the training set. However, in order to avoid data leakage, when performing the tile splitting we should ensure that all the tiles from a single slide go to either the training or the test set. Therefore, from the 6 slides of each tissue, we will choose the slide with the closest number of tiles to 30% of the total tiles to be in the test set, while the remaining five slides will be left for training the model. With this strategy, 5274 tiles (≈74%) were selected for training and 1889 (≈26%) for testing, with the class distribution shown in Table S1. Class imbalances are fixed at the moment of training the model through data augmentation (the procedure is detailed in section S3.2)."

R.2.10: - The deep learning results are an example that tiles processed using PyHIST can achieve high prediction performance, but it doesn't necessarily prove that it is better than other baseline or competing approaches. WSIs from different part of the body can be quite distinguishable, so many different tiling approaches could produce similar results. The Results section could include comparisons of performance and computation time for several tiling methodologies. How does PyHIST stack up against other techniques?
We consider that a benchmark in terms of prediction accuracy is not directly in the scope of PyHIST for three reasons:

i) classification is not the only task that can be performed with image tiles, since they can also be used for other tasks such as unsupervised learning, adversarial learning, etc.,
ii) in the case of classification, different tiling methodologies will produce different sets of input data, therefore, model prediction performance is not directly comparable (at least for the task we solve in the use case) since the sets of data that are being tested are different; such a comparison might only be fair when attributing a label at the slide level rather than at the tile level, and this would require an additional meta-learning step to combine the information from all the tiles that might be too complex for a use-case example; and,

iii) even if we focused only on the classification use case, there are many external factors that can drive prediction performance besides the tiling methodology: model architecture, hardware type, tile size, batch size, data augmentation transformation steps, data sampling, etc.

In this regard, we have now added a clarification note about the use case in the main text: "We note that this classifier is only an exercise to show end-users how to quickly prepare WSI data using PyHIST to generate tiles, reducing the overhead to start performing downstream analyses: further tuning of the model with more data is desirable to ensure that the classifier is robust enough to generalize to different types of unseen WSIs for a real application."

We do, however, believe that it is useful to have an agnostic comparison of tile generation runtimes for the methodologies included within PyHIST, and now provide benchmarking information for all the tiling methods in Supplementary Text S1.

6) Availability and future directions:

R.2.11: - The SVS limitation is mentioned here but should also be addressed earlier in the Intro or Design sections. For example, "PyHIST is currently limited to only SVS format due to/because…".
In this revision, we have performed source code improvements in PyHIST to easily incorporate the features planned for future development. As a consequence, we now experimentally support more WSI formats (Trestle .tif and Hamamatsu .ndpi and .vms slides). This is now detailed at the end of the introduction section:

"PyHIST was developed to process Aperio SVS/TIFF WSIs due to this format being supported by large slide databases such as The Cancer Genome Atlas (TCGA) which has approximately 31,000 WSIs [19] and The Genotype-Tissue Expression Project (GTEx) with approximately 25,000 WSIs [20]. PyHIST currently has experimental support for other formats (see Supplementary Text S1)."

7) Figures:

R.2.12: - Figure 2 A: histology is ambiguous since the top panel for 'T-brain' shows artefactual tissue rather than brain tissue with cell bodies of neurons or glia etc. This is repeated for 3rd, 4th and 5th (from left) T-breast, and 1st (from left) T-colon.
The tiles in this figure were randomly sampled as previously described in the caption. In a classification problem, each tile is assigned a probability of pertaining to each tissue: this means that not all tiles will be representative of the histologic structures present (in the context of cancer,

for this use case) for a given tissue since not all tiles will have a high probability assigned for the ground truth class.

The reviewer correctly observed that neurons were not present in those brain images and that the histologic structure was aberrant. In this use case, the tissue samples correspond to a specific cancer type instead of normal tissue. Glioblastoma (in the brain) is characterized by highly anaplastic cells, necrosis, and microvascular proliferation, and neurons or normal glial cells are not found inside these tumors. The same principle applies to images corresponding to other tissues.

To avoid misinterpretation, we now:

i) Amended the main text to clearly indicate that these are cancer-affected tissues.
ii) Instead of showing random tiles, we show examples of the top 30 tiles (5 per tissue) predicted with the highest confidence (i.e. probability closest to 1 for the ground truth label).
iii) Explicitly show the cancer type in the tile labels (encoded with an abbreviation) in Fig. 2a, S6, S7, Tables S1 and S2, and,
iv) Had the tiles of Figure 2a. inspected by Santiago Rojas, from the Morphological Sciences Department (Human Anatomy and Pathology Units) of the Faculty of Medicine of the Autonomous University of Barcelona, with a strong background in human histology and pathology. With this visual inspection, we corroborated that the characteristics observed in the tiles are compatible with the histopathological diagnosis provided by TCGA. This assessment is now included in Supplementary Text S3.4. We have included Santiago Rojas as an author in our manuscript.

8) Supplementary Materials:

R.2.13: - Section S3: cropping the image tiles is mentioned – what is the size of these crops and are these kept uniform each time? Explanation is required for clarity of the user.
We have now included detailed explanations for the TCGA use case data preparation and model training steps (including data augmentation) in Supplementary Text S3. All the steps and the code are also explained throughout the reproducible notebooks available in PyHIST's documentation webpage. For the specific query about the crop size:

"Data augmentation is performed over the training dataset with the following set of ordered transformations for each tile: a randomly resized crop (from 80% of the original size up to 100%, where no crop is performed) is taken from the 512x512 input tile, a random rotation of in the range of (-15, 15) degrees is performed, the tile can be flipped horizontally with probability 0.5, the tile is then resized to 224x224 (which is the default size for ResNet152), then transformed into a [0, 1]-normalized tensor and finally each pixel is normalized across the color channels using

ImageNet's mean ([0.485, 0.456, 0.406]) and standard deviation ([0.229, 0.224, 0.225]). For the test set, only the last three steps of the transformations are applied (i.e. tiles are resized to 224x224, [0, 1]-normalized tensor, and ImageNet color-normalized)."

R.2.14: - Section S2: the segmentation parameters seem to be an important part of tiling, but it is still unclear how they work. Is this a way of capturing tiles that have background in a certain orientation? How do parameters for border and corners interact with the background percentage and how does this influence segmentation?

We have included a new section (Supplementary Text S2.2) with explanations about the main parameters for graph-based segmentation, which addresses these queries. Visual aids explaining the concepts of these parameters are now available in PyHIST's online documentation. In short: the *--k-const* parameter is a way to define how large detected regions should be to consider them as individual components in the image (a larger value of *k* means detected tissue regions are larger in the mask). The borders and corners parameters are an aid to identify the background color of the mask in graph-based segmentation mode. The *--percentage-bc* parameter defines how large the borders and corners should be: the higher this value, the larger the borders would be to evaluate the background color.
